# Supplementary material for: Gender Influences Gut Microbiota among Patients with Irritable Bowel Syndrome
Source: Int J Mol Sci. 2023 Jun 21;24(13):10424. doi: 10.3390/ijms241310424 (PMC10341582; doi:10.3390/ijms241310424)
Supplement: Supplementary file 1 [file ijms-24-10424-s001.zip › ijms-2437198-supplementary.pdf]

Table S1. Identification of the taxonomic level of type by 16S rRNA NGS sequencing.

Microorganisms - types	IBS	controls
Firmicutes	90.78%	90.93%
Actinobacteria	6.6%	6.45%
Verrucomicrobia	1.25%	0.94%
Tenericutes	0.01%	0.12%
Proteobacteria	0.42%	1.01%
Cyanobacteria	0.05%	0,00%
Synergistetes	0.04%	0,00%
Fusobacteria	0.12%	0.02%
Bacteroidetes	0.01%	0.02%
not identified bacteria	0.54%	0.42%
Euryarchaeota	0.17%	0.09%
not identified archea	0.01%	0.00%

Table S2. Identification of the taxonomic level of order by 16S rRNA NGS sequencing.

Microorganisms - classes	IBS	controls
Clostridia	90.74%	90.86%
Coriobacteriia	4.15%	3.26%
Actinobacteria	2.23%	2.88%
Alphaproteobacteria	0.29%	0.71%
Deltaproteobacteria	0.07%	0.27%
Epsilonproteobacteria	0.05%	0.01%
Gammaproteobacteria	0.01%	0.02%
Chloroplast	0.05%	0,00%
Synergistia	0.04%	0,00%
Fusobacteriia	0.12%	0.02%
Bacteroidia	0.00%	0.01%
Mollicutes	0.01%	0.12%
Methanobacteria	0.17%	0.09%
not identfied	2.07%	1.75%

Table S3. Identification of the taxonomic level of order by 16S rRNA NGS sequencing.

Microorganisms - orders	IBS group	Controls
Clostridiales	90.68%	90.79%
Coriobacteriales	4.15%	3.26%
Actinomycetales	2.23%	2.88%
Rhizobiales	0.19%	0.47%
Rickettsiales	0.01%	0.00%
Desulfuromonadales	0.07%	0.27%
Campylobacterales	0.05%	0.01%
Enterobacterales	0.01%	0.00%
Aeromonadales	0.00%	0.01%
Streptophyta	0.05%	0.00%
Synergistales	0.04%	0.00%
Fusobacteriales	0.12%	0.02%
Acholeplasmatales	0.01%	0.12%
Bacteroidales	0.00%	0.01%
Methanobacteriales	0.17%	0.09%
Not classified	2.22%	2.07%

Table S4. Identification of the taxonomic level of family by 16S rRNA NGS sequencing.

Microorganisms - families	IBS group	Controls
Lachnospiraceae	41.15%	42.44%
Ruminococcaceae	34.67%	35.31%
Peptostreptococcaceae	5.04%	3.76%
Mogibacteriaceae	0.40%	0.31%
Christensenellaceae	0.40%	0.30%
Clostridiaceae	0.96%	0.41%
Tissierellaceae	0.10%	0.00%
Heliobacteriaceae	0.01%	0.05%
Coriobacteriaceae	4.15%	3.26%
Streptomycetaceae	0.25%	0.19%
Thermomonosporaceae	0.01%	0.00%
Campylobacteraceae	0.05%	0.01%
Enterobacteriaceae	0.01%	0.00%
Succinivibrionaceae	0.00%	0.01%
Synergistaceae	0.04%	0.00%
Fusobacteriaceae	0.12%	0.02%
Acholeplasmataceae	0.01%	0.12%
Methanobacteriaceae	0.17%	0.09%
Not identified	12.46%	13.72%

Table S5. Identification of the taxonomic level of genus by 16S rRNA NGS sequencing.

Microorganisms - genera	IBS group	Controls
Ruminococcus	6.54%	5.12%
Oscillospira	5.27%	4.02%
Blautia	14.13%	14.38%
Clostridium	4.30%	5.47%
Shuttleworthia	1.17%	3.62%
Moryella	0.37%	0.73%
Pseudobutyrivibrio	0.17%	0.45%
Peptostreptococcus	0.01%	0.00%
Christensenella	0.02%	0.01%
Anaerovorax	0.17%	0.13%
Mogibacterium	0.23%	0.18%
Clostridium	0.96%	0.41%
Finegoldia	0.01%	0.00%
Gallicola	0.01%	0.00%
Peptoniphilus	0.01%	0.00%
Collinsella	2.52%	2.14%
Streptomyces	0.20%	0.19%
Actinocorallia	0.01%	0.00%
Campylobacter	0.05%	0.01%
Succinivibrio	0.00%	0.01%
Propionigenium	0.12%	0.02%
Candidatus Phytoplasma	0.01%	0.12%
Methanobrevibacter	0.17%	0.09%

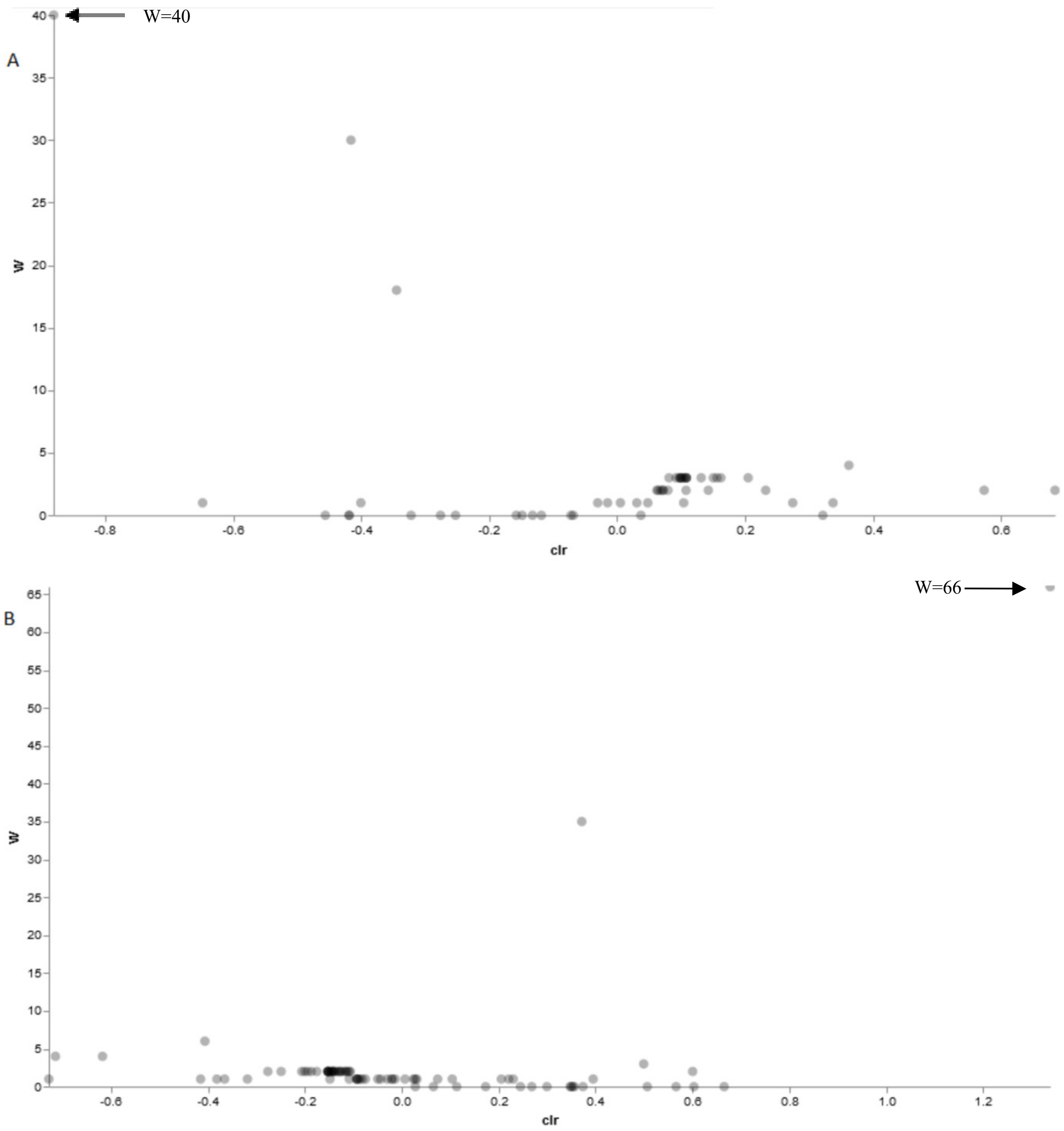

Figure S1. The graphs present the ANCOM analysis for the bacterial component of the gut microbiome based on ASVs in people with IBS and the control group, at the taxonomic levels of (A) family and (B) genus. W represents the number of rejections of the sub-hypothesis for a given (A) family and (B) genus, thus determining statistical significance. clr represents the difference of one group from the average for a specific sequence (ASVs). Statistical significance was observed at taxonomic levels for bacteria of the *Coriobacteriaceae* family (W = 40 for the test at the family level; W = 66 for the analysis at the genus level).

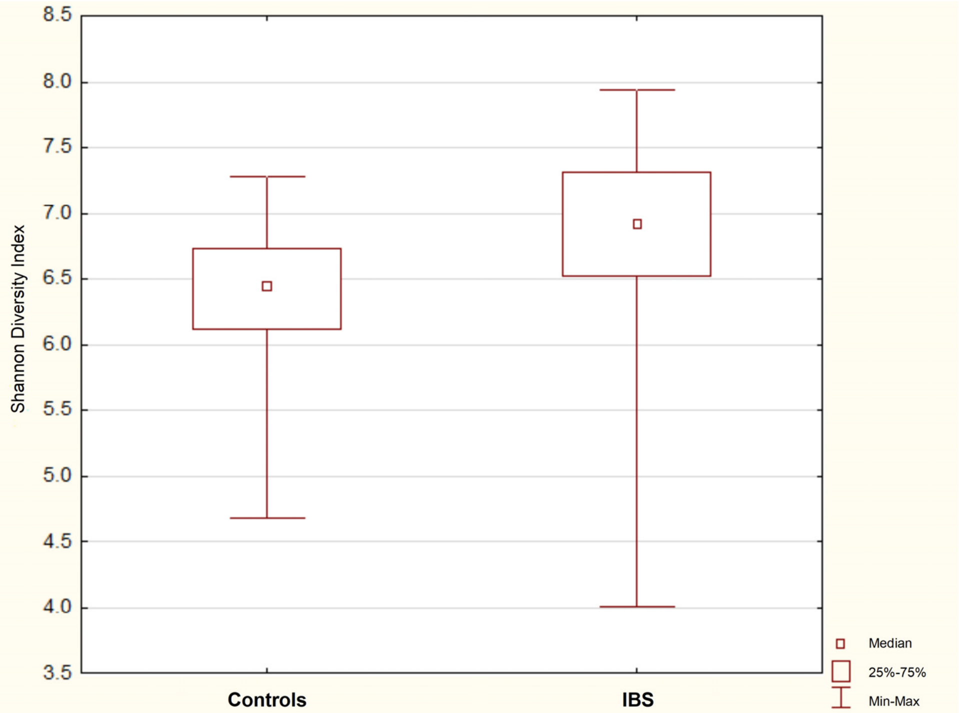

Figure S2. The gut microbiome bacterial element α-diversity among patients with IBS and unaffected individuals. The most diverse was the intestinal microbiota composition among patients with IBS.

Table S6. The bacterial component of gut microbiome of females with irritable bowel syndrome and unaffected females using Dunn's *post hoc* test regarding relative values of ASVs.

	Study groups	N	Me	Min.	Max.	Q1	Q4	p[a]
order *Clostridiales*	I	69	0	0	0.004	0	0	0.019
	II	40	0	0	0	0	0	
Mogibacteriaceae	I	69	0	0	0.007	0	0	0.044
	II	40	0	0	0.003	0	0	
Synergistaceae	I	69	0	0	0.013	0	0	0.036
	II	40	0	0	0.001	0	0	
Blautia spp.	I	69	0.076	0	0.497	0.044	0.149	0.040
	II	40	0.055	0	0.627	0.035	0.076	
Shuttleworthia spp.	I	69	0.008	0	0.084	0.002	0.021	<0.001
	II	40	0.026	0	0.104	0.016	0.055	

I *– female with IBS;* ***II*** *– unaffected female;* ***N*** *– study group size;* ***Me*** *– median;* ***Min.*** *– minimum;* ***Max.*** *– maximum;* ***Q1****– lower quartile;* ***Q3*** *– upper quartile;* [a]*–Dunn post hoc*

Table S7. The bacterial component of the gut microbiome of males with irritable bowel syndrome and unaffected males using Dunn's *post hoc* test regarding relative values of ASVs.

	Study groups	N	Me	Min.	Max.	Q1	Q4	p[a]
Coriobacteriaceae	III	51	0.004	0	0.033	0	0.010	0.020
	IV	30	0	0	0.023	0	0.002	
Shuttleworthia spp.	III	51	0.011	0	0.079	0.002	0.029	0.035
	IV	30	0.036	0	0.128	0.010	0.057	

III *– male with IBS;* ***IV*** *– unaffected male;* ***N*** *– study group size;* ***Me*** *– median;* ***Min.*** *– minimum;* ***Max.*** *– maximum;* ***Q1*** *–lower quartile;* ***Q3****– upper quartile;*[a]*–Dunn post hoc test*

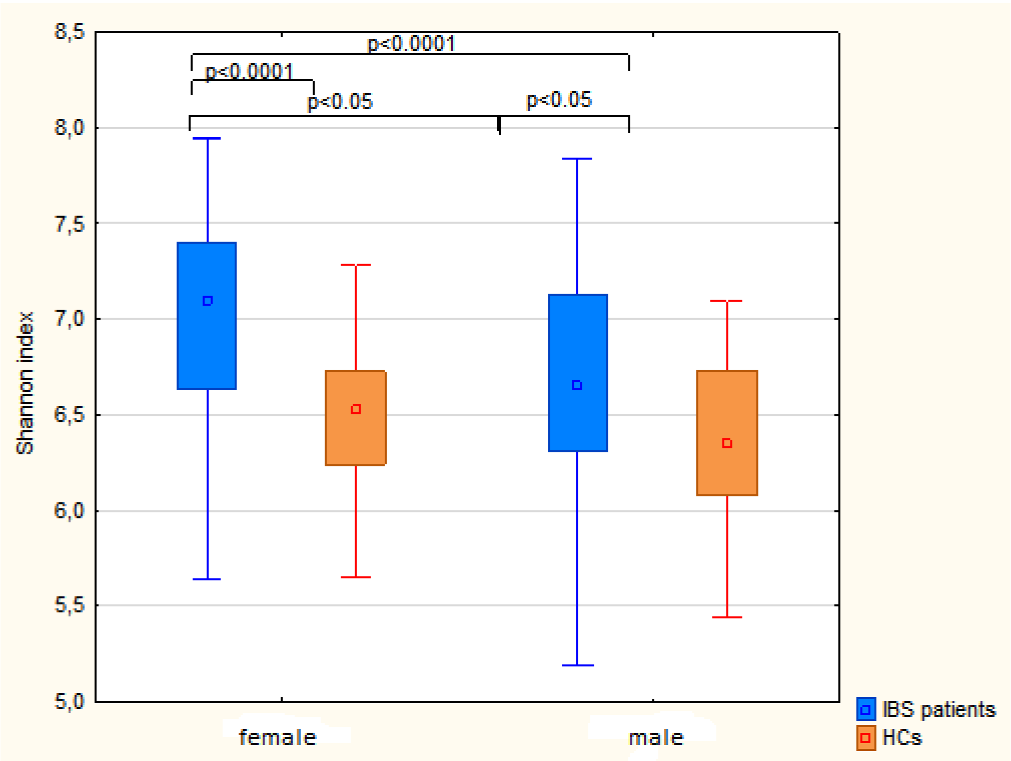

Figure S3. The gut microbiome bacterial element α-diversity among patients with IBS and non-IBS control individuals, taking into account the criterion of gender division. The most diverse was the intestinal microbiota composition among females with IBS. However, the least varied was the gut microbiome of control males.

Table S8. The bacterial component of the gut microbiome of females and males with irritable bowel syndrome using Dunn's *post hoc* test regarding relative values of ASVs.

	Study groups	N	Me	Min.	Max.	Q1	Q4	p[a]
order *Clostridiales*	I	69	0	0	0.004	0	0	0.011
	III	51	0	0	0	0	0	
Synergistaceae	I	69	0	0	0.013	0	0	0.018
	III	51	0	0	0	0	0	

I *– female with IBS;* ***III*** *– male with IBS;* ***N*** *– study group size;* ***Me*** *– median;* ***Min.*** *– minimum;* ***Max.*** *– maximum;* ***Q1*** *– lower quartile;* ***Q3*** *– upper quartile;* [a]*–Dunn post hoc test*

Table S9. Kruskal–Wallis data analysis of *Clostridiales* bacteria concerning IBS types.

IBS patients *Clostridiales* order

gender	disease type *	N	Me	Min.	Max.	Q1	Q4	p[a]
female	D	24	0	0	0.001943	0	0	0.5207
	C	12	0	0	0.004417	0	0	
	M	16	0	0	0.001835	0	0	
	U	1	0	0	0	0	0	
male	D	19	0	0	0	0	0	
	C	8	0	0	0	0	0	
	M	10	0	0	0	0	0	
	U	7	0	0	0	0	0	

** D - IBS-D (diarrhea); C- IBS-C (constipation); M - IBS-M (mixed); U- IBS-U (undefined)*

Table S10. Kruskal–Wallis data analysis of *Synergistaceae* order bacteria concerning IBS types.

IBS patients		*Synergistaceae*						
**gender**	**disease type***	**N**	**Me**	**Min.**	**Max.**	**Q1**	**Q4**	**p[a]**
female	D	24	0	0	0.13401	0	0	0.3140
	C	12	0	0	0.008626	0	0	
	M	16	0	0	0	0	0	
	U	1	0	0	0	0	0	
male	D	19	0	0	0	0	0	
	C	8	0	0	0	0	0	
	M	10	0	0	0	0	0	
	U	7	0	0	0	0	0	

**D - IBS-D (diarrhea); C- IBS-C (constipation); M - IBS-M (mixed); U- IBS-U (undefined)*

Table S11. The effect of several bacterial components on abdominal pain in the last three months in patients with irritable bowel syndrome using Dunn's *post hoc* test regarding relative values of ASVs.

	**Me**	**Min.**	**Max.**	**Q1**	**Q3**	**p[a]**
Blautia spp.	0.150	0	0.615	0.101	0.213	0.481
Mogibacteriaceae	0.002	0	0.013	0	0.004	0.274
Synergistaceae	0	0	0.013	0	0	0.045
Clostridiales	0.925	0.478	0.990	0.900	0.948	0.481

Me *– median;* ***Min.*** *– minimum;* ***Max.*** *– maximum;* ***Q1*** *– lower quartile;* ***Q3*** *– upper quartile; [a]–Dunn post hoc test*
